# Usability Evaluation of a Mobile Phone–Based System for Remote Monitoring and Management of Chemotherapy-Related Side Effects in Cancer Patients: Mixed-Methods Study

**DOI:** 10.2196/10932

**Published:** 2018-12-21

**Authors:** Saeed Moradian, Monika K Krzyzanowska, Roma Maguire, Plinio P Morita, Vishal Kukreti, Jonathan Avery, Geoffrey Liu, Joseph Cafazzo, Doris Howell

**Affiliations:** 1 Department of Supportive Care-Research Division Princess Margaret Cancer Centre University Health Network Toronto, ON Canada; 2 The Lawrence S Bloomberg Faculty of Nursing University of Toronto Toronto, ON Canada; 3 Department of Medical Oncology and Hematology, Princess Margaret Cancer Centre University Health Network Toronto, ON Canada; 4 Institute of Medical Science Faculty of Medicine University of Toronto Toronto, ON Canada; 5 Department of Computer and Information Sciences University of Strathclyde Glasgow United Kingdom; 6 School of Public Health and Health Systems University of Waterloo Waterloo, ON Canada; 7 Institute of Health Policy, Management and Evaluation University of Toronto Toronto, ON Canada; 8 Princess Margaret Cancer Centre University Health Network Toronto, ON Canada; 9 Division of Medical Oncology and Hematology Department of Medicine University of Toronto Toronto, ON Canada; 10 Epidemiology Division Dalla Lana School of Public Health University of Toronto Toronto, ON Canada; 11 Centre for Global eHealth Innovation University Health Network Toronto, ON Canada; 12 Institute of Health Policy, Management & Evaluation University of Toronto Toronto, ON Canada

**Keywords:** mobile apps, mobile health, mobile phone, patient-centered care, patient remote monitoring, self-care, symptom management, usability testing, mobile phone

## Abstract

**Background:**

As most chemotherapy is administered in the outpatient setting, patients are required to manage related side effects at home without direct support from health professionals. The Advanced Symptom Management System (ASyMS) has been developed to facilitate the remote monitoring and management of chemotherapy-related toxicity in patients with cancer, using patient-reported outcomes questionnaires and a clinician alerting system.

**Objective:**

This study aims to evaluate the usability of the ASyMS, a mobile phone–based technology, from the perspective of Canadian patients with cancer receiving chemotherapy to identify existing design, functionality, and usability issues and elicit their views, experiences, and satisfaction with the ASyMS.

**Methods:**

We used a mixed-method approach to data collection with user-based testing, a think-aloud technique, semistructured interviews, and short answer questionnaires with a purposive sample of 10 patients with cancer. Participants attended usability testing sessions at the Centre for Global eHealth Innovation, University Health Network, and performed specific tasks on the ASyMS device. The test was videorecorded and each task was timed during the test. After the usability sessions, participants completed a posttest questionnaire and participated in a semistructured qualitative interview. A thematic analysis was used to code and categorize the identified issues into themes that summarized the type and frequency of occurrence.

**Results:**

The thematic analysis generated 3 overarching themes as follows: ASyMS user-friendliness; usefulness of ASyMS (content quality and richness); and intention to use. Results from the posttest questionnaire indicated that 80% (8/10) of participants had great motivation to use the ASyMS, 70% (7/10) had positive perceptions of the successful use of the ASyMS, and all (10/10, 100%) had a positive attitude toward using the ASyMS in the future. Most identified design and functionality issues were related to the navigation of the ASyMS device and a desire for a more attractive design with advanced functionality and features. The main general design recommendations were as follows: enhance the readability of the screen; implement advance options (eg, search option); and support better navigation.

**Conclusions:**

The ASyMS has shown positive perceptions of patients in usability testing and qualitative interviews. An evaluation of the effects of the ASyMS on symptom outcomes in a clinical trial is needed.

## Introduction

### Background

Systematic chemotherapy continues to be the main treatment modality for almost all major cancer types [1]. Chemotherapy is associated with a myriad of symptoms and adverse treatment side effects that can range from mild to life-threatening, severe, and disabling [2]. Therefore, early recognition and effective management of these symptoms by both clinicians and patients are critical to reducing physical and psychological treatment sequalae [2].

There is growing evidence in support of using patient-reported outcome measures (PROMs) for improving symptom management [3-5]. The increased number of mobile phone users creates opportunities for developing models of supportive care that use these technologies for monitoring PROMs to improve home-based, proactive “real-time” symptom monitoring and management [6-8]. Several Web-enabled PROMs systems have been trialed in oncological settings [4,8-10] and shown to support patients in managing chemotherapy-related symptoms [3,11], improve symptom control [11,12], and enhance patient-clinician communication [4]. However, few of the current systems have been developed in line with best-practice guidelines in user-centered design nor have verified the system usability during the stages of system development [5,13], which could impact their use by patients and clinical integration in practice settings [14].

The aim of this study, which is part of a larger project to enhance the provision of timely, high-quality, person-centered supportive care, is to evaluate the usability of a mobile phone–based technology, the Advanced Symptom Management System (ASyMS), from the perspective of Canadian patients with cancer (colorectal and lymphoma) receiving chemotherapy in a controlled usability testing environment. The secondary aim of our end-user testing is to explore users’ performance and satisfaction with the system interface and their perspectives and experience with the system and the content of ASyMS [9]. In addition, this study assesses the ASyMS against a set of human factors design guidelines and heuristics to increase the likelihood of discovering more design features and function issues that could impact user experience and willingness to use the system.

### Advanced Symptom Management System

The ASyMS, one of the more advanced remote monitoring systems, is a mobile phone–based device designed to monitor and manage chemotherapy-related toxicity in the home setting. It enables real-time remote monitoring of cancer symptoms using PROMs [15]. The ASyMs uses innovative risk prediction modeling and decision-support tools that allow for timely, high-quality, person-centered supportive care for better treatment toxicity management [10,16].

Patients using the ASyMS complete an e-symptom PROMs questionnaire to assess the occurrence, severity, and distress associated with each symptom. After completing the questionnaire, patients immediately receive evidence-based, self-care advice on the mobile phone based on the specific symptoms reported, which facilitates the self-management of symptoms. Leveraging evidence-based algorithms, symptoms reported through the device that meet a threshold criteria (ie, high level of severity) trigger alerts to cancer care clinicians, usually nurses, who on the receipt of an alert can view patients’ symptom reports on a secure webpage and contact patients directly at home by telephone, enabling the initiation of proactive clinical interventions (Multimedia Appendix 1) [17].

**Figure 1:**
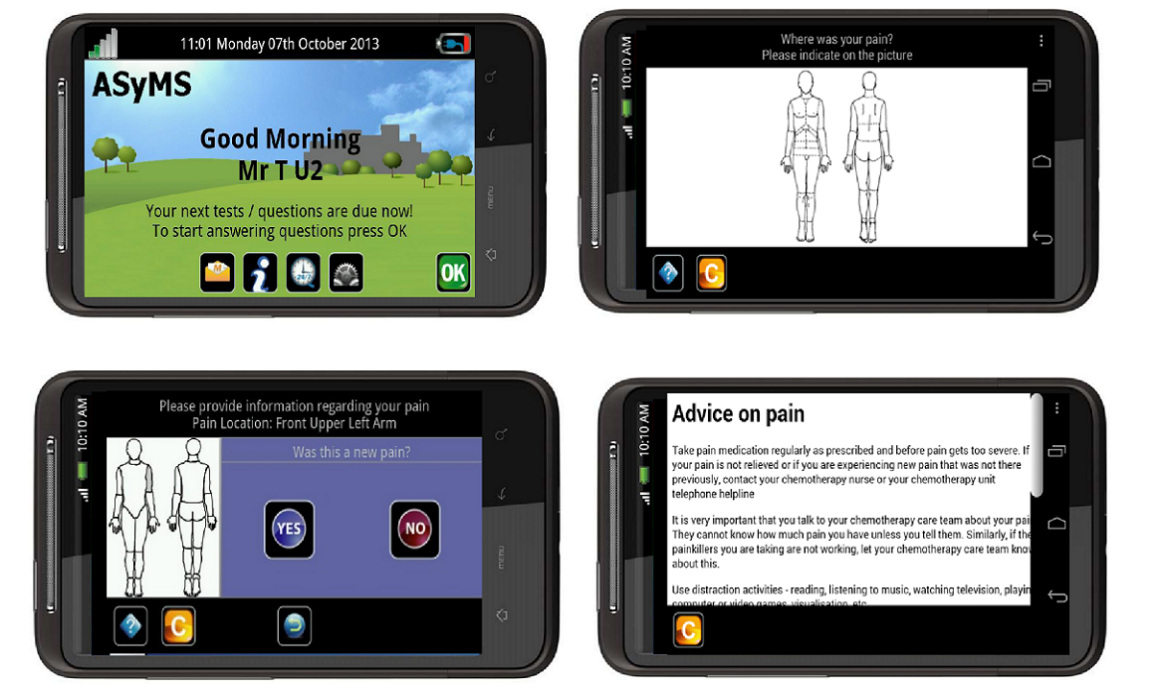
Patient handset screenshots. (Source: Docobo Ltd).

The ASyMS was developed in the United Kingdom based on the extensive patient and clinician engagement, and its utility and acceptability have been tested in UK populations [18,19]. The effect of the ASyMS intervention on patient outcomes is uncertain and is being tested in a large multisite trial in European countries [16]. We undertook a study to test the usability of the ASyMS program to identify its potential for the uptake in a Canadian cancer population. The ASyMS program was installed on an Android mobile phone with a 5.00-inch touchscreen display with a resolution of 720 pixels by 1280 pixels. Figure 1 and Multimedia Appendix 2 show a preview and some features and functions of the ASyMS.

## Methods

### Study Design

We used a mixed-method approach (qualitative and quantitative data sources) to increase the depth of evaluation and support methodological triangulation to improve the reliability and validity of findings [20]. Mixed-methods also allow for a more comprehensive understanding of participants experience and enable the identification of specific usability issues [20,21]. A usability study evaluates how a specific process or product works for individuals and the extent to which a user can use a product to achieve specific goals (interaction between user and task in a defined environment) [22,23].

Data collection combined user-based testing using a think-aloud technique, semistructured qualitative interviews based on a qualitative descriptive methodology [24], and short answer quantitative questionnaires; all these methods have been used widely for usability testing [25]. Specifically, think aloud is a user-related method for assessing usability where users are encouraged to verbalize their perceptions out loud as they interact with the system [26]. Participants’ experiences with the system evaluated through qualitative interviews and questionnaires can inform potential for future uptake [27].

### Participants and Setting

Estimation of the sample size for a usability test depends on several variables, including types of test users available, the mission criticality of a system (any factor that is essential for system operation), and problem discovery rate (the number of usability issues that can be uncovered by users) [28,29]. Although, it has been shown that 80% of the usability problems can be detected with 4 or 5 participants in a usability testing [30], Faulkner [29] found that the minimum percentage of identified usability issues increased from 55% to 82%, and the mean percentage of issues increased from 85% to 95% when the number of participants was increased from 5 to 10. Thus, in this study, we aimed to recruit a minimum of 10 patients. We used a purposive sampling method to ensure maximal variation in end-user characteristics, specifically younger (age <50 years) and older (age >50 years) adult patients with diverse cancer types (colorectal or lymphoma), males and females, and those with and without experience in using mobile technology.

The Institutional Review Board Approval was obtained from the University Health Network (UHN) to conduct the study prior to recruitment (#15-9432). Patients were recruited from ambulatory follow-up clinics at the Princess Margaret Cancer Center, a cancer research center affiliated with the University of Toronto as part of the UHN. The inclusion criteria were that patients received, at least, one cycle of chemotherapy for treatment of their cancer (colorectal or lymphoma), were aged >18, and able to participate in usability testing for “think aloud” in English. All participants gave informed written consent for participation in the study.

### Data Collection

The main goal of user-centered methods is to involve real users, elicit their views and experiences of the intervention to identify usability issues [31,32]. To meet the aim of this study, usability sessions were videorecorded from multiple angles, and participants were encouraged to share their thoughts verbally as they progressed through a set of predefined tasks (think aloud) [26]. We aimed to elicit feedback and identify design, functionality, and usability issues. In addition, participant experiences, thoughts, feelings, and satisfaction with the ASyMS were assessed by an audiotaped, semistructured, face-to-face qualitative interview with participants and through completing a short questionnaire (modified Telehealth Acceptance Measure, TAM), immediately after usability testing sessions (Multimedia Appendix 3).

The TAM questionnaire comprises 10 questions on a Likert scale ranging from 1 to 7; higher scores indicate greater motivation to use telehealth, more favorable perceptions of the successful use of telehealth, greater patients’ belief that significant others would like them to use telehealth, and more positive attitude toward using telehealth. The TAM questionnaire is designed to assess patients’ motivation to use telehealth and includes questions that are derived from the theory of planned behavior, a model that explains the factors that underpin people’s motivation to act [33]. We used this questionnaire to indicate participants’ overall motivation and readiness to use the ASyMS device, assess participants’ perceived behavioral control, subjective norms, attitudes toward the device, and the extent to which individuals perceive that significant others want them to use the device.

### Usability Testing Procedure

Participants attended usability testing sessions at the Healthcare Human Factors labs at the Centre for Global eHealth Innovation, UHN. Each participant was advised that the aim was to test the ASyMS device and not participants. In addition, participants received written and verbal information regarding the testing procedure, and a brief introduction to the ASyMS before usability testing commenced.

Before starting the session, participants completed a demographic questionnaire. Participants were given a case scenario and a simulated symptom experience they might have during one of their chemotherapy cycles. Participants were requested to follow the tasks provided to them on the ASyMS device, representing typical user goals. Throughout testing, each participant was requested to perform specific tasks that consisted of the following: completing the e-symptom questionnaire (PROMs) on the ASyMS device; finding information about side effects and self-care; filling out the anytime section of the symptom questionnaire; and finding a history of side effects (Multimedia Appendix 4). A trained moderator guided participants through the testing procedure but did not intervene or disrupt the thinking-aloud process. Furthermore, from the observation room, behind a one-way mirror, 2 observers watched the interaction, made notes about what was verbalized, and observed to inform the analysis, and ensured the entire session was recorded. Each task was timed during the test.

After the usability sessions, participants completed the posttest questionnaire to assess their perceptions about the usability of the ASyMS (Multimedia Appendix 3). In addition, they participated in a face-to-face interview regarding the utility and acceptability of the ASyMS in managing chemotherapy symptoms, parts of the content or aspects of the system they liked or disliked, and the reason for their response. The complete testing procedure for all steps averaged approximately 2 hours (range 1.5-2 hours).

### Data Analysis

The audio and video recordings from the usability and interview sessions were transcribed. The thematic analysis was used to identify all emerging issues and the relations between the themes [34,35]. The identified issues were coded and categorized according to the type and frequency of occurrence [35]. Data collection and analysis continued until no more patterns or themes were emerging from the data [36]. Two members of the research team reviewed the transcripts. Any discrepancies between reviewers were resolved through discussion or the involvement of a third reviewer, if necessary. All qualitative data were coded using NVivo 10 qualitative data analysis software. In addition, a set of variables related to the participants’ performance, including the number of errors each participant made, requests for help, the time taken to complete the task, participant feedback, observers and moderator’s notes, and reviewing the videos, were used to identify a list of usability issues. Descriptive statistics (means, medians, ranges, frequencies, or percentages) were used to summarize these data.

## Results

### Participant Characteristics

Table 1 presents the characteristics of the study participants. Of 10 participants, 7 were male and 3 were female, with an average age of 68 (range 18-78) years. Most participants (n=8) had higher education (college or university). All participants had their own mobile phone, of which 70% (7/10) had a smartphone, whereas 30% (3/10) owned a regular cell phone (not a smartphone). In addition, 60% (6/10) of participants mentioned that they were comfortable or very comfortable using these devices; 80% (8/10) were comfortable using the internet.

### Quantitative Results

Using the video analysis, the task completion times, the number of errors made by participants while completing tasks, and the number of times they asked for help are shown in Table 2. We followed the TAM developers instructions to score and interpret the TAM (Multimedia Appendix 3). Overall, 80% of participants (8/10) scored >4 on Q2, Q4, and Q5 (mean=5.8), indicating high motivation to use the ASyMS device. In addition, 70% of participants (7/10) scored >4 on Q3, Q6, and Q7 (mean=5.6), indicating they had positive perceptions of the successful use of the ASyMS, and all participants (n=10) scored >4 on Q8 and Q9 (mean=6.1), showing they believed that significant others would like them to use the ASyMS. Furthermore, all participants (n=10) scored ≥5 on Q10 (mean=6.3), suggesting a positive attitude toward using the ASyMS device in future (Table 3).

**Table 1:** Participants’ characteristics (N=10).

Characteristics		Value
Age, median (range)		68 (18-78)
**Sex, n**		
	Male	7
	Female	3
**Education, n**		
	High school	2
	University or college	5
	Postgraduate degree (eg, Doctor of Philosophy)	3
**Own a phone, n**		
	Smartphone	7
	Regular cell phone	3
**Hours use a computer each week, n**		
	Not at all	1
	1-2 h	1
	4-5 h	1
	>7 h	7
**Comfortable using a smartphone, n**		
	Not at all	1
	A little comfortable	3
	Comfortable	4
	Very comfortable	2
**Comfortable using a computer, n**		
	Not at all	N/A^a^
	A little comfortable	4
	Comfortable	3
	Very comfortable	3
**Comfortable using the internet, n**		
	Not at all	N/A
	A little comfortable	2
	Comfortable	4
	Very comfortable	4
**Cancer type, n**		
	Gastrointestinal cancer	3
	Lymphoma	7

^a^N/A: not available.

**Table 2:** Quantitative results (time and SD, errors, and requests for help).

Task	Mean task completion time (SD) in seconds	Frequency of error, *n*_error_ (*n*)^a^	Frequency of requests for help, *n*_help_ (*n*)^b^
Task 1: Complete e-symptom questionnaire	846 (135)	25 (8)	47 (9)
Task 2: Find information about side effects and self-care	502 (250)	52 (9)	32 (8)
Task 3: Filling out anytime questionnaire	232(124)	35 (10)	37 (10)
Task 4: Find history of side effects	257 (70)	34 (10)	26 (9)

^a^*n*_error_ represents the number of times an error was made, and *n* represents the number of people who made the error.

^b^n_help_ represents the number of times a request for help was made, and *n* represents the number of people who made the request for help.

**Table 3:** Telehealth Acceptance Measure: Mean scoring.

Participants’ characteristics				Behavioral intention item		Perceived behavioral control item		Subjective norm item		Attitude item	
No	Age	Sex	Comfortable using a smartphone								
1	68	Female	A little comfortable		7		6.3		7		7
2	78	Male	Not at all		5.7		4.3		5		5
3	18	Male	Very comfortable		6		6.3		5.5		6
4	68	Male	Comfortable		7		6		6		7
5	77	Female	A little comfortable		5.3		3		6.5		6.3
6	59	Male	A little comfortable		3.7		7		7		6.7
7	75	Male	Comfortable		5.3		6		6		5
8	70	Male	Comfortable		7		7		7		7
9	34	Male	Very comfortable		3.7		3		5		6.3
10	55	Female	Comfortable		7		7		6		7

### Qualitative Results

The thematic analysis of the interview transcripts and participants’ feedback generated 3 overarching themes and related subthemes: ASyMS user-friendliness, with subthemes of design, navigation, and ease of use of the ASyMS; usefulness of the ASyMS (content quality and richness), with subthemes of self-care advice and information on the ASyMS, and appropriateness of the ASyMS questions; and intention to use, with subthemes of acceptance and satisfaction with using the ASyMS in future.

### Advanced Symptom Management System User-Friendliness

Both the quantitative and qualitative data from the usability testing identified several design and functionality issues for the ASyMS’s device that may negatively impact its efficient use. Each of the recognized issues was mapped to source events (ie, participants’ feedback, errors, and moderator observation). Moreover, each of the issues was classified in one of the 8 usability heuristics for mobile devices (ie, match between system and the real world, ease of input, and screen readability) [37].

The identified issues shown in Table 4 mostly relate to the navigation of the ASyMS device.

**Table 4:** Identified usability issues.

Problem	Category (usability heuristics)	Source
Introduction screen not intuitive nor informative enough	Match between system and the real world	Feedback; Errors; Request for help
Small screen or font size	Screen readability	Feedback; Observations
Lack of effective color scheme	Screen readability	Feedback; Observations
Lack of advance options (eg, search option)	Consistency and mapping	Feedback; Observations
No option to (send a message) chat with a clinician	Ease of input	Feedback; Observations
Problem with editing and no obvious go back option	Ease of input and Consistency and mapping	Feedback; Errors; Request for help; Observations

Participants through usability testing and the interview commented on the need for a more advanced and attractive design, with better functionality and features in ASyMS to better address the needs of end users, as indicated below:

Finding where everything is, it’s not labeled, so it would be easier if every option was labeled...I think it needs a higher-level menu, which may have to be categorized, which allows me to navigate around through it easily.

Participant 1

...add a search button. Having a search button just kills so much time. You can access the entire database in like 2 seconds. Saves a lot of time...I wish it had a search button.

Participant 3

Some older participants (age>65) commented that they would prefer to use ASyMS on a device with a larger screen (with larger font size) and a more effective color scheme that better draws users’ attention toward specific elements on the screen.

A bigger screen for people who need glasses...someone like me whose vision is affected needs a bigger screen

Participant 4

It is always nice for someone in my age if got a bigger text...Of course, if you could make it bigger, would be great.

Participant 7

I have an iPhone, which the text isn’t very much bigger than that, right? Like the text is the same basically. But the screen colors, like this screen color to me (shows iPhone) is a lot easier to read than this (shows the ASyMS’s handset).

Participant 5

As participants were not familiar with the system prior to the usability session and no tutorial of the ASyMS and its functionalities was given, they often felt insecure about their actions and asked for assistance and approval before performing tasks. Most participants mentioned that they would need some time to learn and get familiar with the ASyMS device before they could start to use it regularly. By the end of the usability sessions, when participants had gained some experience with using ASyMS, all participants agreed that with experience in using the ASyMS, they would get familiar with its features and definitely use it more efficiently, as noted in the following participant quote:

If people use this a few times they will be able to (use and) navigate it easily.

Participant 6

### Usefulness of the Advanced Symptom Management System

Regarding the self-care advice and information provided in the library, almost every participant commented that the ASyMS provides a lot of quality information. Two participants suggested that using the information would be easier if the self-care information was better categorized.

The information section, I would rather have that more generalized and categorized. It is easier for the person to use.

Participant 6

There is no easy way to find information here. You have to read the entire list to get what you want, and probably read more than once. Categorization would improve that.

Participant 2

One older (aged 70 years) participant, who felt comfortable using a smartphone and had a high level of education suggested that it would be helpful if the information and advice could be customized to the specific therapy that patients receive. In addition, he commented that the self-care information he sought was not covered in the self-care advice as he experienced different treatment toxicities and suggested to modify and enhance the self-care advice.

It would help if you could customize to the individual...the specific therapy they are receiving...There is one major thing, that is the food. One of the problems in my experience, I went through two different regimens of chemotherapy, different elements of the regimens have different food requirements, some for example do not allow caffeine or alcohol or meat products or spices. None of that is here, that would be helpful if me and my wife are about to contemplate dinner and it tells us can I eat such and such, those answers would be helpful in terms of my chemotherapy. This app tells us about chemotherapy in general, whereas different patients have different regimens of chemotherapy...

Participant 8

### Intention to Use

All participants mentioned that the ASyMS would be a valuable device to use for managing cancer treatment side effects. Results from the modified TAM and the interviews suggested that almost all participants were satisfied and pleased with their experience in using the ASyMS device, and this positively influenced their attitude toward using the ASyMS in the future. Participants indicated confidence that the problem of communication with their health care provider can be solved (to some extent) by using such a device, and it will help participants to manage their symptoms quicker than the other current available options.

Clinician is typically in a hurry, not using a lot of words, sometimes very technical words...easy to get snowed...I always try to bring my wife or my son or my daughter, so they can hear what the doctor says and also ask questions. The conversation between me and my doctor is brief and complex. If I have any questions later, I cannot reach them...I call their secretary, who asks that clinician, and gets back to me in a few days...makes it impossible to ask follow-up questions. There is a problem with the nature of this communication, which a system like ASyMS could improve, if it provided for multiple interactions...I can see value in such a system if it provided that capability.

Participant 10

it actually would give you a pretty good history. And probably you would be able to deal with the nasty symptoms quicker than the other options which is trying to contact your doctor or nurse, and its not that easy.

Participant 2

## Discussion

Despite the proliferative use of mobile technologies in health care, few Web-enabled PROMs systems have been developed with consideration of their quality through comprehensive and rigorous usability evaluations [38]. While ASyMS has undergone several years of development [17,18], this study has added to the knowledge about usability issues, acceptability, and the potential for the uptake of this mobile technology in Canadian cancer populations to manage the acute effects of cancer treatment.The main general design recommendations (according to usability heuristics) for enhancing features of the ASyMS are as follows: enhance the readability and glanceability of screen; implement advance options (including search option, easy identifiable back option, intuitive pop-up screen option, and advanced navigation options, eg, swiping screens, for expert users); and support navigation by creating an option to customize main menu features, particularly the self-care advice to make it easier to find rather than reading through lengthy text.

Concerns have been raised in the literature that modern technologies, such as mobile devices, may not be entirely appropriate for use by all cancer populations, as it might be considered difficult to use. For example, older adults may experience difficulties when using technologies such as mobile devices or smartphones [39]. However, there has been a growing interest in the design of technologies, including innovative health technology design for older adults, who often manage complex health conditions and multiple chronic illnesses, to provide better and more sufficient supportive care services [40]. Our study findings demonstrated that older participants (>65) were interested and had a positive attitude toward using the ASyMS device, although a few of them mentioned that they prefer to use the ASyMS on a larger device with larger font size. Furthermore, they mentioned that their performance was affected by age-related physical and mental health status. This is also shown in previous research that older adults are interested and capable of using modern technologies for managing health care issues [41-43].

Furthermore, limited experience with aspects of mobile technology did not affect the acceptance of the mobile device in this study. This is also consistent with the result of a recent literature review indicating that mobile devices, such as smartphones, can be ideal tools for novice users who have very little understanding of how software or a system in general works, as users learn how to use a touchscreen after a few tries [39]. Although none of the participants had previous experience in using the ASyMS device, all of them became proficient during or by the end of usability testing sessions, indicating that the training period does not need to be long; nevertheless, the incorporation of tutorials and training are important to reduce the time needed by users to learn how to use the system [44,45]. The training should focus on the system features that are more problematic, challenging, and complex for users [43], ensuring that patients feel confident in the use of the system.

Besides design issues and problems observed in the usability testing, participants also commented on the ASyMS content to enable self-management of treatment symptoms. Previous research has shown that a higher perception of the content richness in a system has resulted in a higher perception of the usefulness of the system [46]. The content richness is defined as the adequacy of resources that users can access to improve their activity on a particular technology [47]. As noted by Lee and Lehto [48], the content richness is a key significant predictor of the perceived usefulness [49]. Our findings support a need to enhance the self-care advice and personalized tailoring to treatment regimens to better support patients in taking the required actions for symptom self-management. Evidence-based guidelines for symptom management have been developed [50] and best practices in presenting information in an “actionable” format should be considered in the future design of the ASyMS device [51]. Furthermore, as patients have different learning styles, the use of an extensive library of written, audio, and video information resources and patient education materials and guidelines for symptom self-management would be beneficial.

Although the usability considers a combination of factors (including intuitive design, ease of learning, efficiency of use, memorability, error frequency, and user satisfaction [52,53]), one usability evaluation cannot claim to cover all possible and critical usage situations that can possibly occur. Testing the ASyMS in a real-world setting and evaluation of the effectiveness through a trial is needed given high variability in practices [43,54]. We have customized some of the features in the ASyMS device based on the data derived from this study. For instance, we have modified the content of the self-care library to be more action-oriented to foster patient self-management. Currently, a feasibility randomized controlled trial is under way (NCT03335189) that will identify the implementation and context-related issues prior to a larger, multisite randomized controlled trial in Canada.

## Appendix

Multimedia Appendix 1 ASyMS Monitoring System.

Multimedia Appendix 2 Features of ASyMS.

Multimedia Appendix 3 ASYMS Acceptance Measure.

Multimedia Appendix 4 Usability test script.

## Abbreviations

ASyMS: Advanced Symptom Management System
PROM: patient-reported outcome measure
TAM: Telehealth Acceptance Measure
UHN: University Health Network
