# Supplementary figures and images for: Usability Evaluation of a Mobile Phone–Based System for Remote Monitoring and Management of Chemotherapy-Related Side Effects in Cancer Patients: Mixed-Methods Study
Source: JMIR Cancer. 2018 Dec 21;4(2):e10932. doi: 10.2196/10932 (PMC6320433; doi:10.2196/10932)

# ASyMS monitoring system

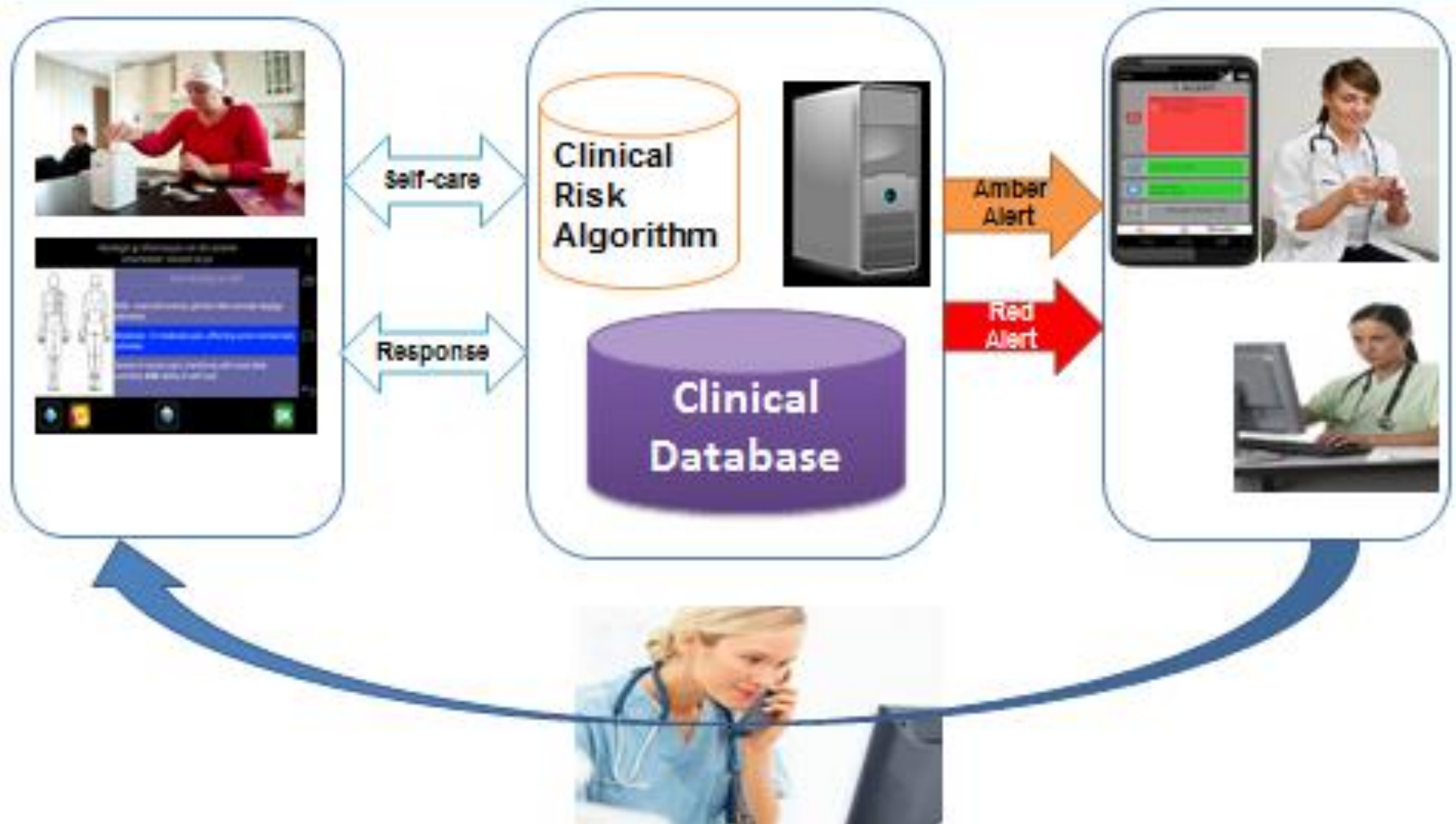

Supplement: Multimedia Appendix 1 [file cancer_v4i2e10932_app1.pdf]
