# Supplementary material for: Usability Evaluation of a Mobile Phone–Based System for Remote Monitoring and Management of Chemotherapy-Related Side Effects in Cancer Patients: Mixed-Methods Study
Source: JMIR Cancer. 2018 Dec 21;4(2):e10932. doi: 10.2196/10932 (PMC6320433; doi:10.2196/10932)
Supplement: Multimedia Appendix 2 [file cancer_v4i2e10932_app2.pdf]

Appendix 2: Some features and functions of ASyMS Handset

The Home Screen

The functions of each of the icons/pictures are described briefly below.

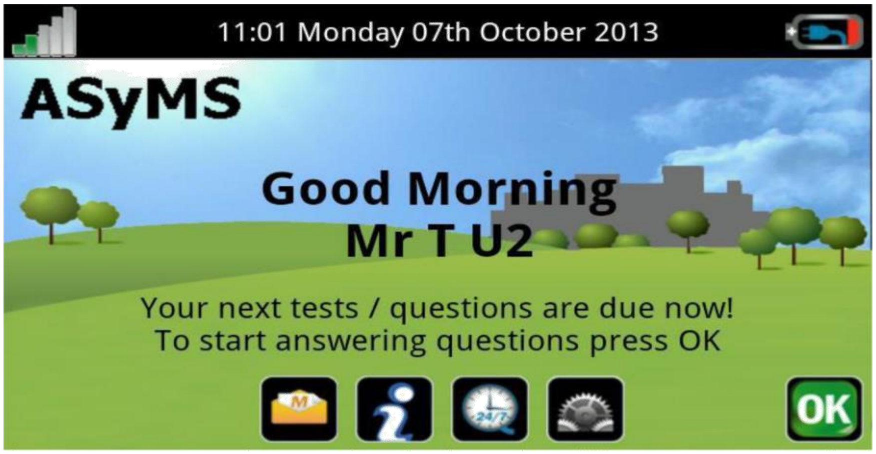

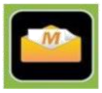

Text Message ICON: If patient receive a message from the clinical care team it will flash. To read the message patient should simply select the icon and the message will be displayed.

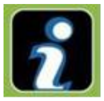

Information ICON: will give patient access to his/her **Symptoms Graph**, the **Self Care Library** and **Useful Contacts.**

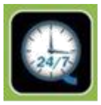

Anytime Questions ICON: will enable patient to answer her/his symptoms questionnaire at any time you feel unwell.

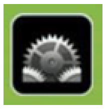

Settings ICON: This is where patient can change the screen brightness, handset volume, and if required, patient can select a visually impaired option. The "Extended Settings" option is for researchers only to enable them to make adjustments to the device during the study if needed.

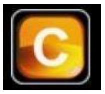

C ICON: is used if you wish to cancel an action or go back to a previous screen.

The next section explains how each of the different functions listed within the Home Screen work and how to navigate between them.

Completing a questionnaire

There are 2 ways to begin entering answers, you can either answer your daily timed questions by tapping ‘OK’ on the home screen, or you can answer ‘Anytime’ questions by tapping the ‘24/7 Clock’ icon on the home screen.

Working through the questionnaire should only take you a couple of minutes.

At the beginning of each cycle you will receive a message asking if your next chemotherapy cycle has started. Press “Yes” if you have started your next cycle, otherwise press “No” (e.g. if your next chemotherapy cycle has been deferred).

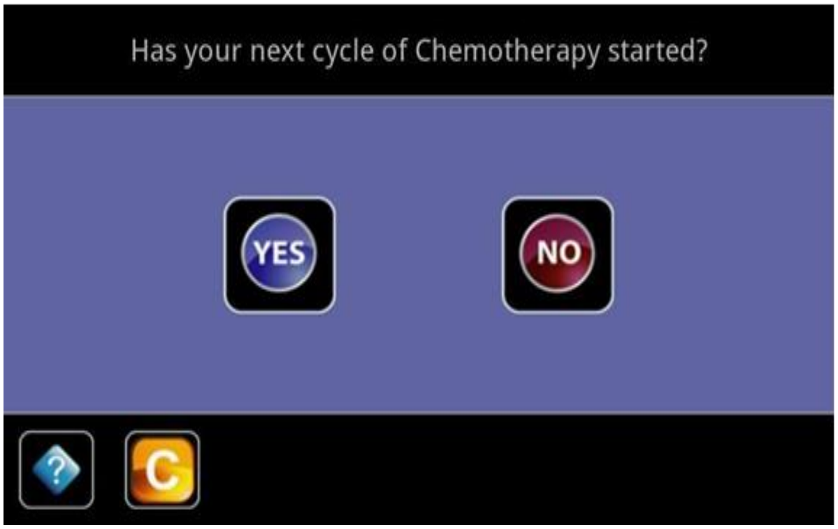

You will be asked whether you are in hospital (as an in-patient) or not. While you are in hospital please continue to fill out your questionnaires. No alerts will be triggered while you are in hospital.

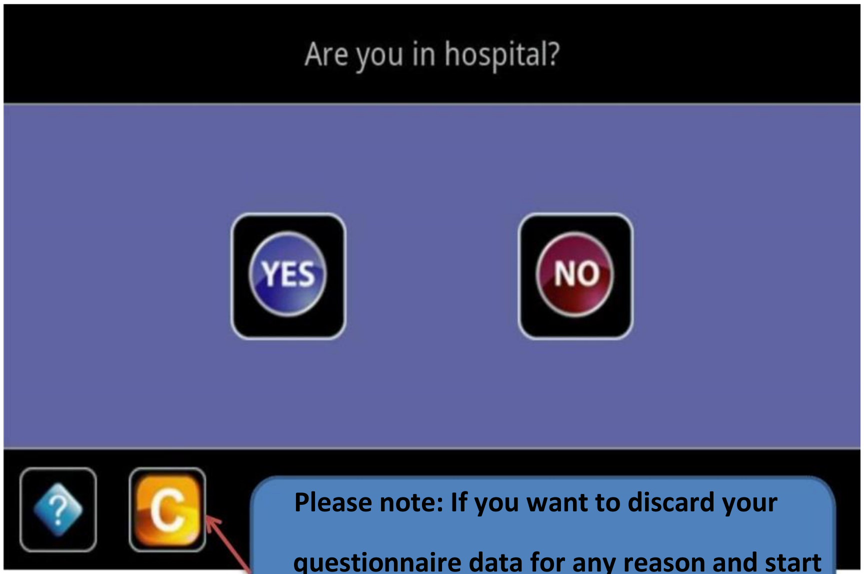

Please note: If you want to discard your questionnaire data for any reason and start again - you can just press the 'C' button.
