# Supplementary material for: Usability Evaluation of a Mobile Phone–Based System for Remote Monitoring and Management of Chemotherapy-Related Side Effects in Cancer Patients: Mixed-Methods Study
Source: JMIR Cancer. 2018 Dec 21;4(2):e10932. doi: 10.2196/10932 (PMC6320433; doi:10.2196/10932)
Supplement: Multimedia Appendix 3 [file cancer_v4i2e10932_app3.pdf]

Appendix 3: ASYMS Acceptance Measure

(For patients participating in usability test)

To be completed by participants

These questions are asking you for your thoughts and feelings about ASYMS.

Please circle the number which represents how you feel about each statement.

1- I intend to use ASYMS								
Definitely do not	1	2	3	4	5	6	7	Definitely do
2- I believe I have the ability to use ASYMS								
Definitely do not	1	2	3	4	5	6	7	Definitely do
3- I want to use ASYMS								
Definitely do not	1	2	3	4	5	6	7	Definitely do
4- How likely is it that you will use ASYMS?								
Very unlikely	1	2	3	4	5	6	7	Very likely
5- My using ASYMS is/ would be…								
Difficult	1	2	3	4	5	6	7	Easy
6- How confident are you that you will be able to use ASYMS?								
Not very confident	1	2	3	4	5	6	7	Very confident
7- Most people who are important to me approve/ would approve of my using ASYMS								
Strongly disagree	1	2	3	4	5	6	7	Strongly agree
8- People who are important to me want me to use ASYMS								
Strongly disagree	1	2	3	4	5	6	7	Strongly agree
9- The ASyMS mobile device will help me manage my chemotherapy toxicities more effectively								
Strongly disagree	1	2	3	4	5	6	7	Strongly agree
10- I found the information and self-care advice that I have received to be worthwhile.								
Strongly disagree	1	2	3	4	5	6	7	Strongly agree
11- I would recommend this system to be used for patients receiving chemotherapy.								

Strongly disagree	1	2	3	4	5	6	7	Strongly agree
12- Overall, my attitude towards using ASYMS is: *(please circle one number on each line)*								
Negative	1	2	3	4	5	6	7	Positive
Anti	1	2	3	4	5	6	7	Pro
Bad	1	2	3	4	5	6	7	Good
