# Supplementary material for: Usability Evaluation of a Mobile Phone–Based System for Remote Monitoring and Management of Chemotherapy-Related Side Effects in Cancer Patients: Mixed-Methods Study
Source: JMIR Cancer. 2018 Dec 21;4(2):e10932. doi: 10.2196/10932 (PMC6320433; doi:10.2196/10932)
Supplement: Multimedia Appendix 4 [file cancer_v4i2e10932_app4.pdf]

Appendix 4: Usability test script for ASyMS-Canada

Hi, ___________. My name is ___________, and I'm going to be walking you through this session today. Before we begin, I have some information for you, and I'm going to read it to make sure that I cover everything. You probably already have a good idea of why we asked you here, but let me go over it again briefly.

We are testing a new device called the Advanced Symptom Management System, or ASyMS for short. This Symptom Management System is a new smartphone application (show the patient a smartphone), and we are testing to see just how easy it is for our patients to use the smartphone application for recording any symptoms they might be experiencing. This study is currently in its most early stage of development in Canada, therefore we are looking to just have a small sample of our patients to use this application so we can have a sense of its practicality, accessibility, usefulness, and any problems it might have. The session should take about *45 minutes to one hour.*

The first thing I want to make clear right away is that we're testing the device, not you. You can't do anything wrong here. In fact, this is probably the one place today where you don't have to worry about making mistakes.

As you use the device, I'm going to ask you as much as possible to try to think out loud: to say what you're looking at, what you're trying to do, and what you're thinking. This will be a big help to us.

Also, please don't worry that you're going to hurt our feelings. We're doing this to improve this app, so we need to hear your honest reactions.

If you have any questions as we go along, just ask them. I may not be able to answer them right away, since we're interested in how people do when they don't have someone sitting next to them to help. But if you still have any questions when we're done I'll try to answer them then. And if you need to take a break at any point, just let me know.

You may have noticed the microphone and some video cameras around you. With your permission, we're going to record what happens on the screen and our conversation. The recording will only be used to help us figure out how to improve the app, and it won't be seen by anyone except the people working on this project. And it helps me, because I don't have to take as many notes. The camera will record the phone screen and not your face, it means that the camera focus on this paper area (show the paper area to the patient) so please work with and keep the phone in this area.

Also, there are a few people from the research team observing this session in another room.

Do you have any questions so far?

OK. Before we look at the phone, I'd like to ask you to complete these forms please if you didn't before.

☐ Complete demographic information &clinical baseline forms.

Before we start, I'm going to give you the opportunity to play with the device so you can familiarize yourself with it. So feel free to pick it, turn it on and push as many buttons on the device as you'd like. Please explore freely (2-3 minutes)

☐ Allow this to continue for two or three minutes, at most.

Now that you've had a chance to play with the device, I am going to start asking you some questions. And please remember, to try to think out loud: to say what you're looking at, what you're trying to do, and what you're thinking. To begin, I'm going to ask you to look at the screen and tell me what you make of it: what strikes you about it, what you can do here, and what it's for. Just look around.

Thanks. Now I'm going to ask you to try doing some specific tasks. I'm going to read each one out loud.

☐ Hand the participant the first scenario, and read it aloud.<br>☐ Allow the user to proceed until you don't feel like it's producing any value or the user becomes very frustrated.<br>☐ Repeat for each task or until time runs out

We will ask you to answer the symptom questionnaire based on the scenario that I give you to answer the questions. Based on this case scenario you may cover all the different questions listed in the symptom questionnaire.

When you are doing these tasks please verbalize your perceptions of using the app and your experience navigating the device. You should tell me what you are thinking! It will

feel strange but this is very important OK?

TASKS:

Task 1:

We're going to start at the home page. Your first task is to answer the questions. So please start answering questions.

What do you think about these questions?

Probes:

- Number of questions?
- Wording of questions?
- Navigation?

By the way, I have just noticed that we made a mistake in our scenario; actually you have experienced *nausea* or ………. for the last *24 hours* ……… and not *diarrhea*………... Can you back and correct your answer accordingly.

What are you thinking you should do here? Please don't forget to think out loud.

☐ Allow to correct the answers for two minutes, at most.

How was that task?

Task 2:

There are some information about side effects and self care/ general information about chemotherapy/ feelings and emotions. Can find information about changes to sense of taste/ eating well/ your mouth/ constipation…….

Remember to say what you're thinking!

What do you think? Is that easy to find the information?

Can you read it easily?

Do you think this information is useful?

What about the font, typestyle, color? Is that readable?

Now please find information about feelings after cancer treatment.

Again! Please remember to say what you're thinking!

Task3:

You already fill out the questionnaire and answer all the questions this morning; but in the afternoon you feel unwell and would like to send extra information by filling out another questionnaire , Please ignore the OK (as it is mistaking show up here in the screen). Can you start ?

Can find where to start?

Ok, thanks. Can you cancel that and back to home screen?

Task 4:

Imagine you want to check the history of your pain with your care provider. Please find this information.

Please remember to say what you're thinking!

What do you think? Is that easy to find the information?

Currently there is no symptoms graph in your app; but can you tell us what you think about this kind of data?

Is that understandable for you?

Is that meaningful to you?

Tell me about your thinking

Many thanks for all you work today. You've been really helpful. I hope you enjoyed it!

If you'll excuse me for a minute, I'm just going to see if the people on the team have any follow-up questions they'd like me to ask you.

☐ Ask if the observers have any questions and then probe anything you want to follow up on..

Now please complete this short questionnaire that last approximately 5 minutes.

☐ Complete ASYMS Acceptance Measure.

Now that you have used ASyMS, we are interested in learning about what you liked and disliked about the system. So, I ask you some questions about your experience using ASYMS. It takes about 20-30 minutes and asking questions regarding your thoughts, feelings and satisfaction with ASyMS. Is that OK?

☐ Use Semi-Structured Interview Guide

1. How easy was it to use/understand the ASyMS to monitor and manage the toxicities of cancer treatment?

Probes: What did you find easy to understand about ASyMS? Getting advice to facilitates self-management of symptoms? *What were the challenges of navigating through the ASyMS? What would make it easier to navigate through the ASyMS?* Can you tell me more about that?

2. What did you find hard to use/understand about the ASyMS?

Probes: Wording of questions? Completing the e-symptom questionnaire? Sending this information? Receiving self-care advice? Can you tell me more about that?

3. What did you like best about the ASyMS?

Probes: Design of the ASyMS, *Information, layout, navigation, sending message, receiving message, tagging message for timing of response*

4. What did you like least about the ASyMS?

Probes: Probes: Design of the ASyMS, *Information, layout, navigation, sending message, receiving message, tagging message for timing of response*

5. What would you like to change about the ASyMS?

Probes: Number of questions? Wording of questions and/or self-care advice? Can you tell me more about that?

6. What would you like to add to the ASyMS that would help you to better manage the toxicities of chemotherapy ?

Probes: Can you tell me more about that? Can you give me an example?

7. Is there anything else you would like to tell us about the ASyMS?

Probes: Can you tell me more about that?

Thank you so much for time and helping us with this study.

- [ ] Stop the screen recorder and save the file.
- [ ] Give them their incentive.
- [ ] Thank them and escort them out.
